# Tr14 gel for the treatment of acute ankle sprains: a plain language summary of the TRAUMED trial

**DOI:** 10.57264/cer-2025-0018

**Published:** 2025-06-05

**Authors:** Ludger Gerdesmeyer, Helmut Pabst, Konstantin Cesnulevicius, Myron Schultz, Alta Smit, Gino Kerkhoffs

**Affiliations:** 1 Orthopedics & Trauma Surgery, Kiel Municipal Hospital, Kiel, Germany; 2 Dr. med. Helmut Pabst Private Practice, Gilching, Germany; 3 Heel GmbH, Baden-Baden, Germany; 4 Department of Orthopedic Surgery & Sports Medicine, Amsterdam Movement Sciences, Amsterdam University Medical Centers, University of Amsterdam, Amsterdam, The Netherlands

**Keywords:** acute ankle sprain, diclofenac, plain language summary, TRAUMED

## Abstract

**What is this summary about?:**

This is a summary of an article discussing the results of the TRAUMED trial, which was originally published in the *Journal of Clinical Medicine*. This trial studied how well Tr14 gel helped with ankle sprains compared with either a dummy treatment (placebo) or a common painkiller called diclofenac.

**What were the results of this trial, and what do they mean?:**

The results of this trial showed that Tr14 was effective at reducing pain caused by an acute or sudden ankle sprain compared with placebo and worked just as well as diclofenac. The results also suggested that Tr14 led to faster pain relief and improved foot and ankle function compared with placebo and was at least as effective as diclofenac.

**Clinical Trial Registration:** NCT06192420

**This is an abstract of the Plain Language Summary of Publication article.**

To read the full Plain Language Summary of this article, click here to view the PDF.

